# Transdiscal Versus Cement-Augmented Pedicle Screw Fixation for Thoracolumbar Fractures in Patients with Diffuse Idiopathic Skeletal Hyperostosis (DISH)

**DOI:** 10.3390/diagnostics16213576

**Published:** 2026-10-29

**Authors:** Akiyoshi Miyamoto, Masato Tanaka, Ahmed Majid Heydar, Tadashi Komatsubara, Ryo Ugawa, Masakazu Nagamatsu, Mitsuru Yagi, Haruki Funao, Norihiro Isogai

**Affiliations:** 1 Department of Orthopaedic Surgery, Okayama Rosai Hospital, 1-10-25 Chikkō Midorimachi, Minami Ward, Okayama 702-8055, Japan; hello.akkun.11.136@icloud.com (A.M.); dr.a.heydar@gmail.com (A.M.H.); t.komatsubara1982@gmail.com (T.K.); ryougawa0412@herb.ocn.ne.jp (R.U.); 2 Department of Orthopaedic Surgery, International University of Health and Welfare, Narita Hospital, Chiba 286-8520, Japan; yagiman@gmail.com (M.Y.); hfunao@yahoo.co.jp (H.F.); n.isogai@live.jp (N.I.); 3 Department of Orthopaedic and Traumatology Clinic, Memorial Bahçeliever Hospital, Istanbul 34180, Turkey; 4 Department of Radiology, Okayama Rosai Hospital, 1-10-25 Chikkō Midorimachi, Minami Ward, Okayama 702-8055, Japan; n.masa1987215@gmail.com

**Keywords:** diffuse idiopathic skeletal hyperostosis, transdiscal screw, cement-augmented fenestrated pedicle screw, screw loosening

## Abstract

**Background/Objectives:** Rigid internal fixation and early mobilization using pedicle screws have become a standard treatment option for vertebral fractures in patients with diffuse idiopathic skeletal hyperostosis. Poor bone quality and a long lever arm may result in a high rate of screw loosening, screw pullout, and implant failure in these patients. Therefore, researchers have developed various techniques to enhance fixation strength, including cement-augmented fenestrated pedicle screw (CAFPS) and transdiscal screw (TDS) fixation. This study compared the clinical and radiological outcomes of CAFPS and TDS fixation techniques for treating diffuse idiopathic skeletal hyperostosis (DISH)-associated thoracolumbar vertebral fractures. This study compared the clinical and radiological outcomes of CAFPS and TDS fixation techniques for DISH-associated thoracolumbar vertebral fractures. **Methods:** Retrospective comparative cohort study. An evaluation was conducted of patients who underwent posterior instrumentation for vertebral fractures accompanied by DISH, using CAFPS or TDS techniques, at our hospital between August 2019 and March 2025. We assessed preoperative, operative, and postoperative data, outcomes, and radiographic parameters. **Results:** We included 40 patients; 11 received CAFPS, and 29 received TDS fixation. Operative time (104 ± 19.6 min vs. 115 ± 29.4 min, *p* = 0.32), blood loss (87.7 ± 69.4 mL vs. 88.3 ± 64.8 mL, *p* = 0.95), and bone union rate (100% vs. 89.7%, *p* = 0.27) did not differ significantly. The incidence of screw loosening number was higher in the TDS group (5.68% vs. 13.4%, *p* = 0.049). However, patient-level analysis showed no difference (4/11 vs. 7/29, *p* = 0.455). Neither group had implant backouts, failures, or reoperations. **Conclusions:** Transdiscal screws and transdiscal and cement-augmented pedicle screws are useful techniques for treating thoracolumbar vertebral fractures in patients with DISH. These techniques can reduce the level of fixation in vulnerable patient populations.

## 1. Introduction

Diffuse idiopathic skeletal hyperostosis (DISH), the leading cause of ankylosing spinal disorder [1], is characterized by pathological autofusion within vertebral bodies, resulting in long, rigidly fused spinal segments [1]. Because of their reduced flexibility, these segments act as long lever arms that concentrate stress locally rather than distributing it across mobile segments and adjacent ligaments. These abnormal biomechanics increase the ankylosing spine’s susceptibility to vertebral fractures, even after minor trauma [2]. The majority of these fractures occur within the fused segments, resulting in highly unstable three-column spinal injuries [3], which are associated with a significantly elevated risk of spinal cord injury [4] and nonunion [5].

In addition to the abnormal spinal biomechanics induced by DISH, which is considered an independent risk factor for vertebral fractures [6], several studies indicate that low bone mineral density further elevates the risk of vertebral fractures in individuals with DISH [7,8]. Given that the incidence of osteoporosis-related morbidity is significantly higher among the elderly, vertebral fractures associated with DISH predominantly occur within this demographic. As the population of older individuals continues to grow, the prevalence of vertebral fractures in patients with DISH is anticipated to increase.

The prevailing belief is that conservative management of spine fractures in patients with DISH leads to higher rates of morbidity and mortality, which explains the common practice of opting for surgical intervention [9]. Rigid posterior instrumentation and early mobilization using pedicle screws are standard treatment approaches for DISH-associated fractures [3]. Given the long leverage arms of multilevel fused spinal columns and the compromised bone quality in these patients, long-segment instrumentation spanning at least three levels above and below the fracture site is used [3]. Nevertheless, the effectiveness of pedicle screws in osteoporotic spines is diminished, leading to frequent complications, including screw loosening, screw pullout, and implant failure [10]. To address these issues, cement-augmented fenestrated pedicle screws (CAFPS) [11] and transdiscal screws (TDS) [12] have been proposed as surgical alternatives. However, few studies report outcomes for these techniques [13,14], and no clinical studies have compared the two methods for fixing vertebral fractures in patients with DISH. Therefore, this study aimed to compare the clinical and radiological outcomes of CAFPS and TDS for fixing vertebral fractures associated with DISH.

## 2. Methods

### 2.1. Research Design

Our institution’s ethics committee in Okayama, Japan, approved this retrospective cohort study (No. 629, Okayama Rosai Hospital). We obtained informed consent from all patients who underwent the surgical procedures in this study. We identified patients through institutional operation logs and obtained radiographic data from digital imaging archives. We retrospectively assessed patients who underwent posterior fixation for vertebral fractures with DISH using the CAFPS or TDS techniques at our hospital between August 2019 and March 2025. The inclusion criteria for this study were as follows: (1) presence of vertebral fractures, (2) presence of DISH at the fracture level, and (3) patients who underwent posterior fixation using CAFPS or TDS. The exclusion criteria were (1) use of traditional trajectory screws, (2) absence of DISH, (3) follow-up period of less than six months, (4) spinal infection, and (5) history of spinal tumor.

We used the DISH criteria reported by Resnisk [15].

1. Flowing ossification: Presence of flowing calcification/ossification along the anterior or anterolateral aspect of at least 4 contiguous vertebral bodies.
2. Preservation of disc Spaces: Relative preservation of intervertebral disc height in the involved segments, with an absence of extensive underlying degenerative disc disease.
3. Absence of inflammatory joint disease/ankylosis.

For language editing, Grammarly (version v. 1. 2. 298. 1959) was used. The study was conducted in accordance with the Declaration of Helsinki and approved by the Institutional Ethics Committee of Okayama Rosai Hospital (protocol code 629, July 1st, 2026).

### 2.2. Radiological and Clinical Assessments

**Figure 1:**
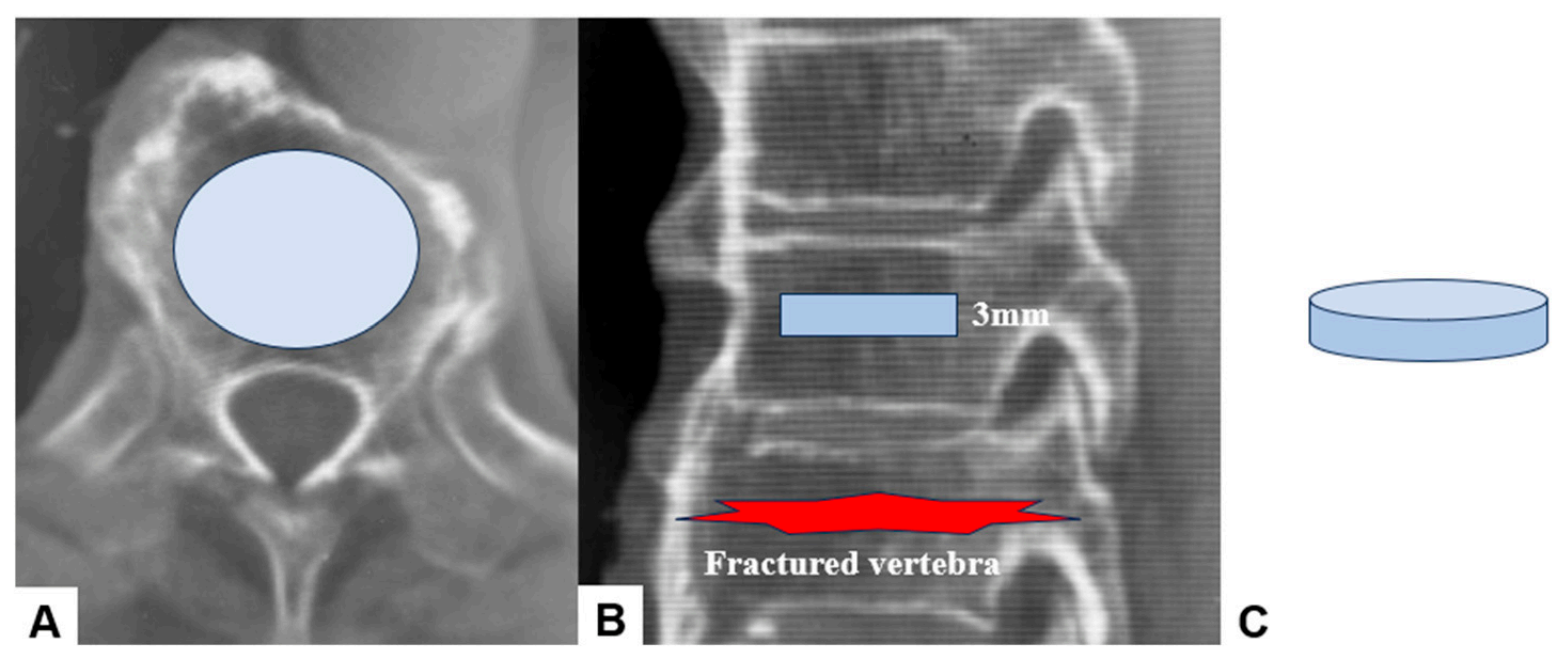
Measurement of HU. (**A**) Axial CT; (**B**) Sagittal reconstruction CT; (**C**) Region of interest (ROI). In the axial CT image, draw a maximum ellipse without touching the cortex.

We defined loosening as a radiolucency >1 mm around the screws, whereas pullout was identified by a halo sign and axial displacement of the screw relative to the immediate postoperative images [16]. Bony union was detected by trabecular bone bridging and by dynamic radiographs, as reported by the senior authors (M.T. and T.K.). In addition, we measured the bone mineral density of one cranial vertebra from the fracture site using CT in Hounsfield Units (HU) with Synapse Vincent (Fujifilm, Tokyo, Japan; Figure 1). Clinically, we checked operative time, blood loss, and reoperation rate in the electronic medical record.

### 2.3. Surgical Procedures

We placed the patient in the prone position on a Jackson frame. Surgery was performed using neuromonitoring. We positioned the navigation reference frame at the spinous process near the fracture level. We positioned the O-arm, performed a CT scan, and transferred the data to the Stealth Station Navigation System Spine 7R (Medtronic Sofamor Danek, Minneapolis, MN, USA).

**Figure 2:**
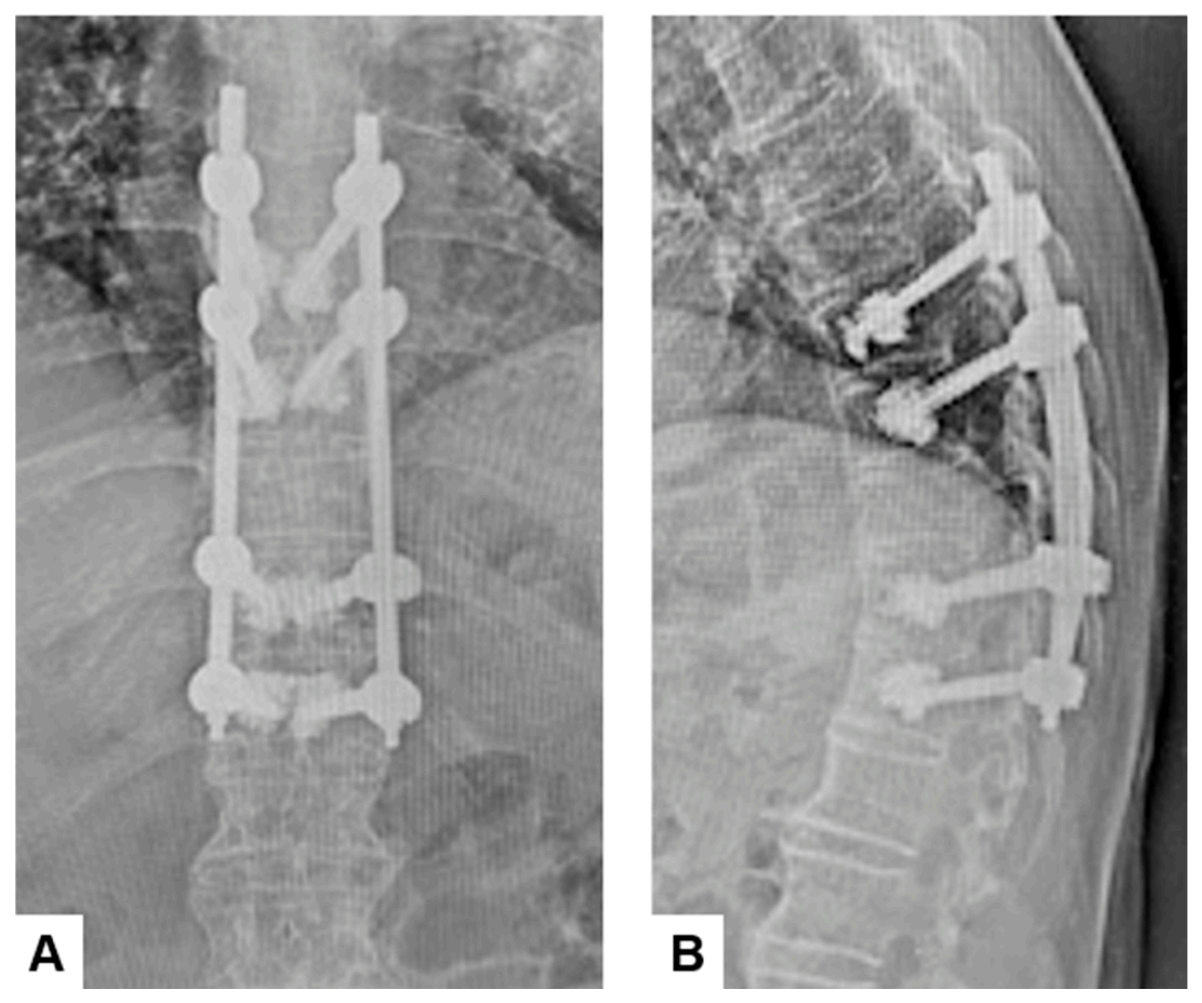
Postoperative anteroposterior and lateral radiographic views treated by posterior fixation with cement-augmented fenestrated pedicle screws for DISH-associated T10 fracture. (**A**) Anteroposterior radiogram; (**B**) Lateral radiogram.

In the CAFPS group, screws were inserted percutaneously two levels above and two levels below the fractured vertebra using the traditional trajectory, and polymethylmethacrylate bone cement was carefully injected into the screws under C-arm control to prevent leakage until the tip was completely filled (Figure 2).

**Figure 3:**
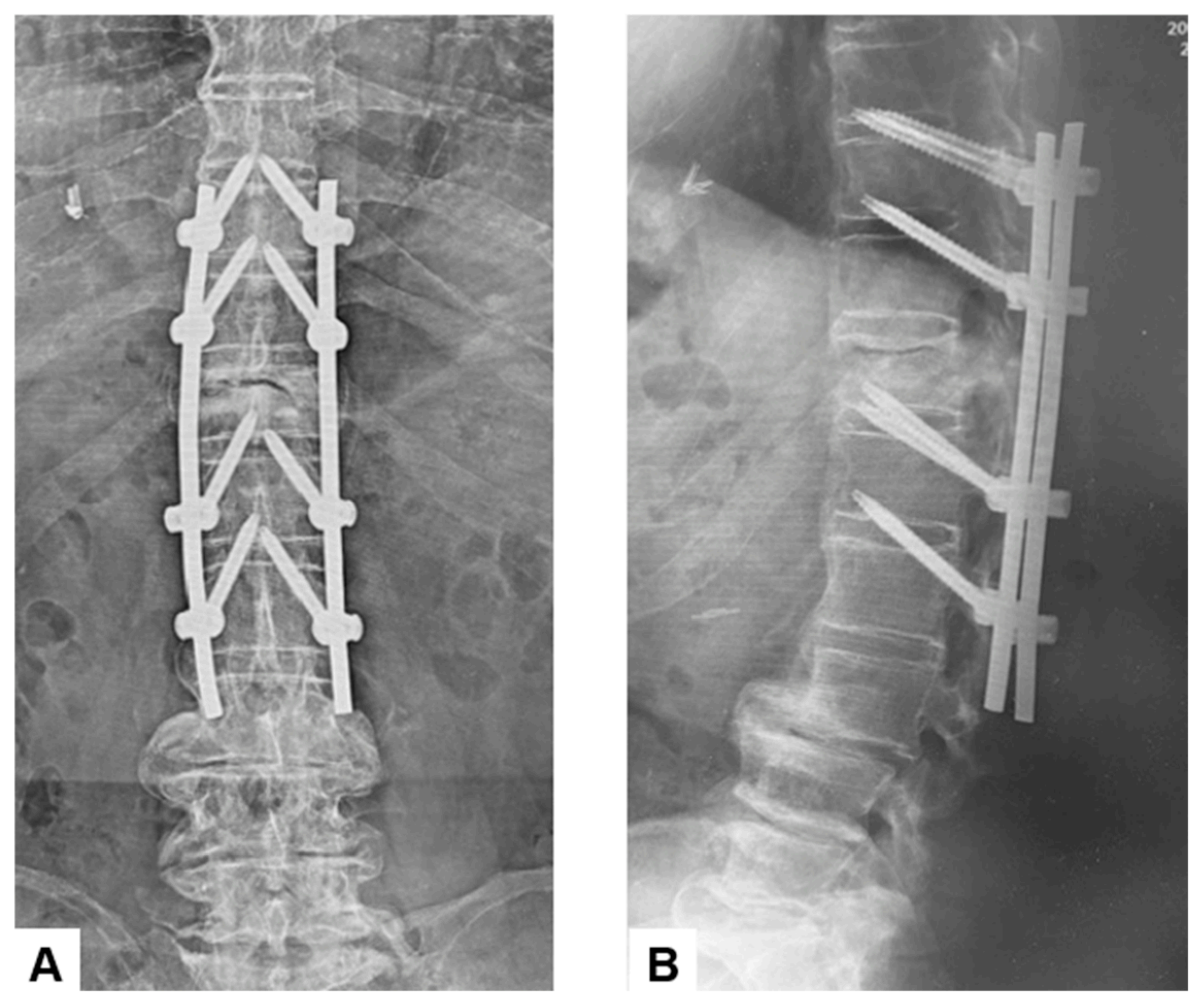
Postoperative anteroposterior and lateral radiographic views treated by posterior fixation with transdiscal screws for DISH-associated T12 fracture. (**A**) Anteroposterior radiogram; (**B**) Lateral radiogram.

In the TDS group, the entry point was positioned just beneath the pedicle, with the screw trajectory extending cranially through the pedicle. This path enabled the screw to penetrate adjacent endplates of two vertebrae: the proximal endplate of the vertebra where the screw was inserted and the distal endplate of the proximal vertebral body (Figure 3). The construct spanned two pedicles above and two pedicles below the fractured vertebra.

### 2.4. Statistical Evaluation

To compare the groups, this study used the Mann–Whitney U test, Fisher’s exact test, and the chi-squared test. Statistical significance was set at *p* < 0.05.

## 3. Results

### 3.1. Patient and Surgical Factors

**Figure 4:**
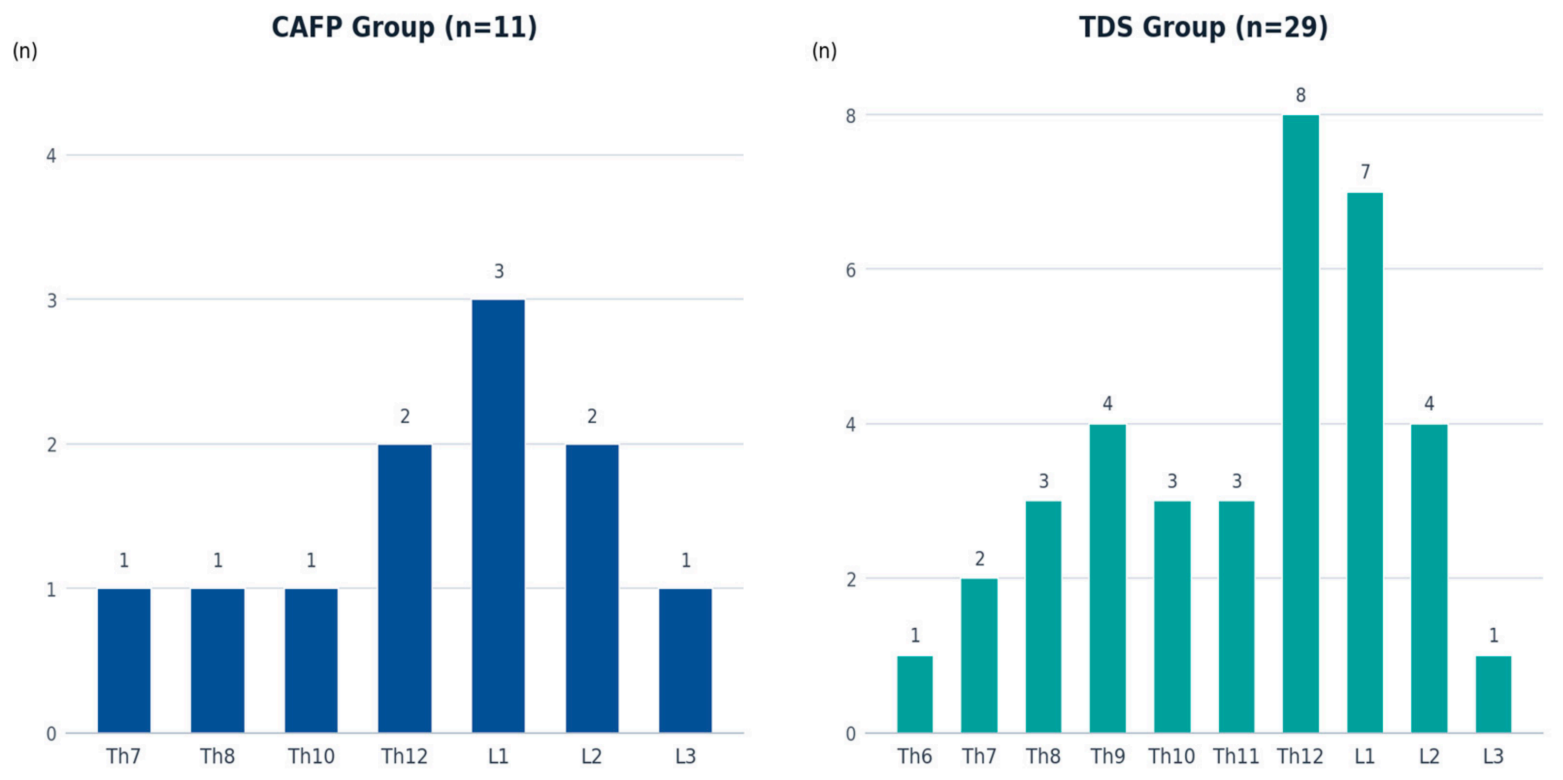
Fracture level of both groups. CAFP: cement-augmented fenestrated pedicle screw, TDS: transdiscal screw.

The study included 40 patients: 11 received CAFPS, and 29 received TDS. The CAFPS group used 88 screws, whereas the TDS group required 232 screws. Baseline characteristics were similar between the groups: the mean age was 81.6 ± 9.6 years in the CAFPS group and 79.7 ± 10.6 years in the TDS group (*p* = 0.54), and sex distribution did not differ significantly (*p* = 0.66). Additionally, the distribution of fracture levels was similar between the CAFPS and TDS groups, with thoracic fractures at 45% versus 55% and lumbar fractures at 67% versus 33% (*p* = 0.20), respectively (Figure 4). Table 1 summarizes the patients’ demographics.

### 3.2. Clinical Results

Operative duration did not differ significantly between the CAFPS and TDS groups (104 ± 20 min and 115 ± 29 min, respectively; *p* = 0.32). Similarly, blood loss was comparable between the two groups (88 ± 69 mL in the CAFPS group and 88 ± 65 mL in the TDS group; *p* = 0.95). Neither group had complications or reoperations.

### 3.3. Radiological Results

**Figure 5:**
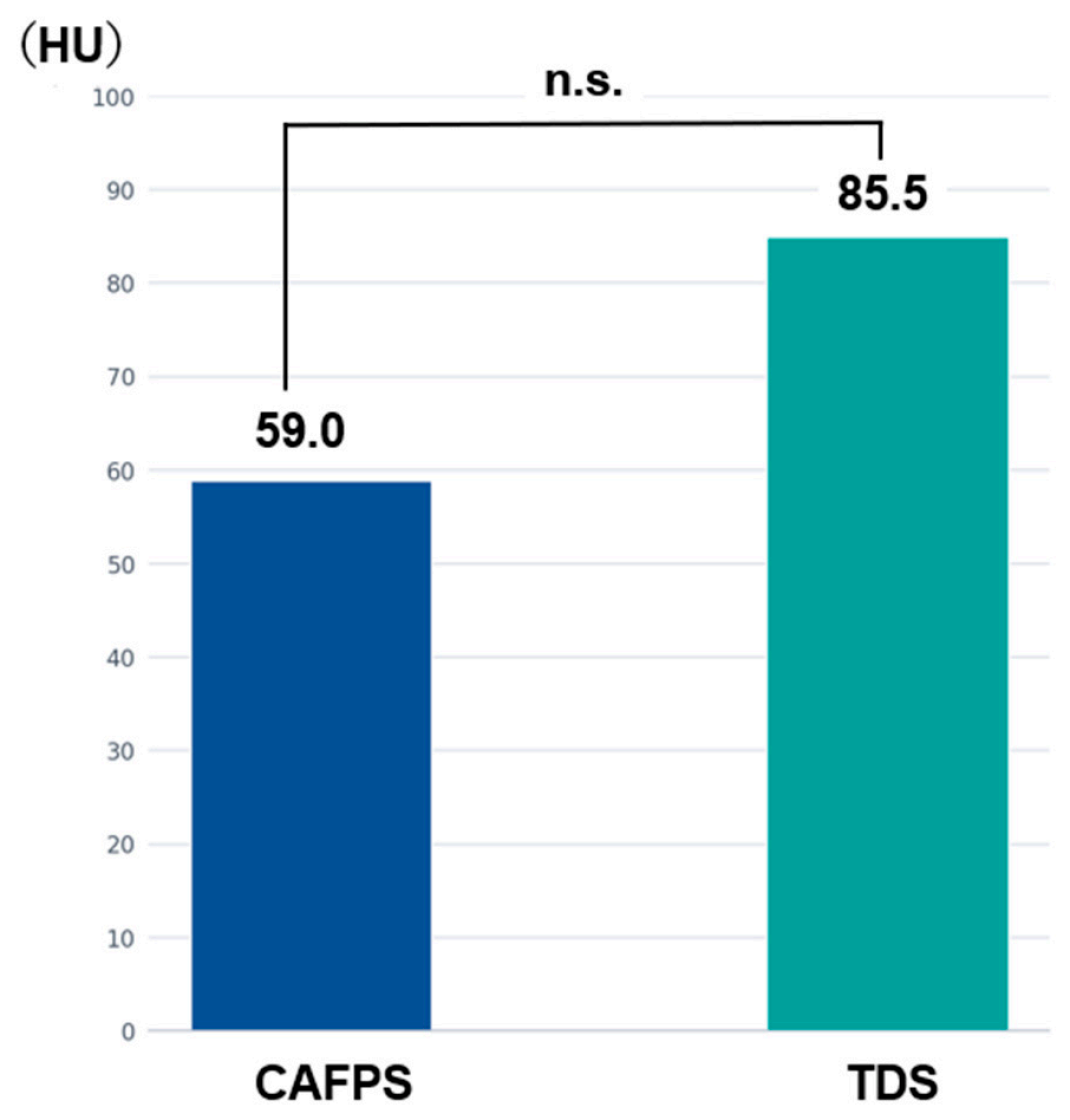
Hounsfield unit of both groups. CAFP: cement-augmented fenestrated pedicle screw; TDS: transdiscal screw, n.s.: not significant.

The incidence of screw loosening was significantly lower in the CAFPS group (5.68%) than in the TDS group (13.4%) (*p* = 0.049). Screw loosening cases did not differ significantly between the two groups. All patients in the CAFPS group achieved bone union (100%), and 26 patients (89.7%) in the TDS group, with no statistically significant difference (*p* = 0.27). Neither group reported additional surgery or implant back-out, as detailed in Table 2. The Hounsfield Unit (HU) values were also comparable (*p* = 0.47) (Figure 5).

## 4. Discussion

In this study, we examined the radiological and clinical outcomes of vertebral fractures in patients with DISH treated with either cement-augmented pedicle screw fixation or transdiscal screw fixation. Although screw loosening was more common in the transdiscal fixation group, both groups achieved good pain relief without reoperation. The surgical risk for patients with DISH-associated vertebral fractures is significantly elevated compared to that of the general population due to their advanced age and multiple medical comorbidities. Conventionally, these patients undergo operative management through open instrumented fusion. Nonetheless, standard open techniques frequently result in considerable morbidity, including substantial blood loss, prolonged operative duration, and an increased incidence of wound-related complications [17]. Consequently, minimally invasive spinal surgery has become increasingly popular for managing these patients [18]. Numerous reports indicate that even when posterior fixation spans an extended vertebral segment, anticipated blood loss during surgery and the incidence of perioperative complications are reduced compared with traditional open surgery [19].

Two factors mainly affect the effectiveness of pedicle screw fixation: bone quality and local loads. In patients with ankylosing spine fractures, recent studies have increasingly documented instances of screw pullout and loosening within a fixation segment that extends up to two vertebrae above and below the fracture site, which can lead to construct failure. Consequently, most studies recommend pedicle screw fixation spanning at least 3 vertebrae above and below the fractured vertebra [20,21]. Expanding the number of instrumented vertebrae inevitably increases the surgical invasiveness, operative duration, blood loss, and perioperative stress. These factors are associated with elevated postoperative complication rates in elderly and medically frail populations. Studies on DISH-associated fractures have consistently reported high perioperative morbidity and mortality rates following extensive posterior instrumentation [22]. Therefore, although long-segment fixation enhances stability, the extent of instrumentation must be judiciously balanced against the heightened perioperative burden of larger constructs [23].

Evidence suggests that enhanced fixation strategies, including cement-augmented pedicle screws and transdiscal screw fixation, can substantially increase construct stability in osteoporotic and ankylosed spines, allowing shorter fixation constructs while maintaining adequate biomechanical strength. Cement augmentation significantly improves pedicle screw anchorage, pullout strength, and resistance to loosening in poor-quality bones, reducing the risk of implant failure and increasing overall construct rigidity [23,24]. Biomechanical and clinical studies show that augmentation techniques can compensate for reduced fixation strength in shorter constructs by improving screw–bone interface stability. Similarly, transdiscal screws traverse adjacent vertebral endplates and engage multiple vertebral bodies, providing a longer screw purchase and superior resistance to pullout and rotational forces compared to conventional pedicle screws [14,25]. These characteristics are particularly advantageous in osteoporotic fractures and ankylosed spinal disorders, where enhanced fixation may reduce the number of instrumented levels without compromising stability or fusion outcomes.

Researchers have recently explored dynamic spinal stabilization as an alternative to conventional rigid fixation in selected patients with osteoporotic vertebral fractures (OVFs), with the rationale that it provides controlled segmental motion while reducing implant-related stress [26,27,28]. However, in a biomechanical cadaveric study, Hoppe et al. demonstrated greater pedicle screw-head displacement with dynamic compared with standard rigid stabilization configurations in osteoporotic vertebrae, whereas cement augmentation significantly improved screw stability in compromised bone [29]. These findings should not be generalized to fractures occurring in an ankylosed spine. Unlike OVFs, fractures associated with DISH often involve a significantly ossified spinal segment that functions biomechanically as a long lever arm. This configuration creates large bending moments and increases the risk of displacement, nonunion, and subsequent neurological deterioration. Consequently, dynamic constructs intended to allow controlled motion may not provide adequate resistance at the fracture site and may increase demands on the screw–bone interface, especially when bone quality is compromised. Therefore, although dynamic stabilization is an emerging option for certain osteoporotic fractures, its efficacy in managing unstable DISH-associated fractures remains unproven, and rigid fixation continues to be the more suitable biomechanical approach.

In our study, no patients experienced serious postoperative complications or fatalities during the follow-up period. The incidence of screw loosening number was higher in the TDS group (5.68% vs. 13.4%, *p* = 0.049). However, patient-level analysis showed no difference (4/11 vs. 7/29, *p* = 0.455). Bone union did not differ significantly between the two groups (100% vs. 89.7%, *p* = 0.27), and neither group required additional surgery or experienced implant back-out. Although screw loosening was more prevalent in the TDS group, patients remained asymptomatic; therefore, clinical outcomes did not differ significantly between the two groups.

This study has several limitations. First, the random assignment of participants to the CAFPS and TDS groups was not feasible. From August 2019 to November 2023, TDS was employed in all cases of vertebral fractures with DISH. However, from December 2023 to March 2025, surgeons’ preferences determined the choice between TDS and CAFPS. Second, the relatively small sizes of both the CAFPS and TDS cohorts restricted the statistical power to detect modest differences in clinical outcomes. Lastly, to determine whether CAFPS or TDS is superior, researchers should increase sample sizes in both groups and conduct long-term follow-up.

## 5. Conclusions

Transdiscal screws and transdiscal and cement-augmented pedicle screws are useful techniques for treating thoracolumbar vertebral fractures in patients with DISH. These techniques (two above- and two below-fixation with TDS or CAFPS) can reduce the fixation level in vulnerable patient populations.

## Figures and Tables

**Table 1:** Patients’ demographics.

	CAFPS Group (n = 11)	TDS Group (n = 29)
Age (years old: Median)	83 (53–89)	82 (55–96)
Gender (Female/male)	5/6	11/18
Comorbidities	HT 2, DM 5	HT 4, DM 6, CKD 1, COPD 1, BD
Neurological status (ASIA)	D 2, E 9	D 5, E 24
Fracture AO classification	B1 1, B3 10	B1 3, B3 26
Osteoporosis treatment (+/−)	7/4	20/9
Follow-up (months: Median)	15.5 (12.0–24.4)	17.1 (12.1–37.2)

CAFP: cement-augmented fenestrated pedicle screw; TDS: transdiscal screw; HT: Hypertension; DM: Diabetes mellitus; CKD: Chronic renal failure; BD: Bipolar disorder; ASIA: American Spinal Injury Association impairment score; AO: Arbeitsgemeinschaft für Osteosynthesefragen.

**Table 2:** Radiological and clinical results of both groups.

	CAFPS Group (n = 11)	TDS Group (n = 29)	*p* Value	Odds Ratio	95% CI	Statistical Test
Screw loosening (+/total)	5/88	31/232	0.049*	0.39	0.15–1.04	Chi-squared test
Case loosening (+/n)	4/11	7/29	0.455	1.71	0.38–7.66	Fisher’s exact test
Boney union (+/n)	11/11	26/29	0.548	N.E.	0.99–1.26	Fisher’s exact test
Implant failure	None	None				
Reoperation	None	None				

CAFP: cement-augmented fenestrated pedicle screw; TDS: transdiscal screw; N.E.: not estimable.

## Data Availability

The data presented in this study are available in the article.
